# The effectiveness of eugenol against cisplatin-induced ototoxicity^☆^

**DOI:** 10.1016/j.bjorl.2018.07.007

**Published:** 2018-08-07

**Authors:** Muhammed Sedat Sakat, Korhan Kilic, Fazile Nur Ekinci Akdemir, Serkan Yildirim, Gizem Eser, Ahmet Kiziltunc

**Affiliations:** a Ataturk University, Faculty of Medicine, Department of Otorhinolaryngology, Erzurum, Turkey; b Agri Ibrahim Cecen University, High School of Health, Department of Nutrition and Dietetics, Agri, Turkey; c Ataturk University, Faculty of Veterinary, Department of Pathology, Erzurum, Turkey; d Ataturk University, Faculty of Medicine, Department of Biochemistry, Erzurum, Turkey

**Keywords:** Cisplatin-induced ototoxicity, Eugenol, DPOAE, Oxidative stress, 8-Hydroxy-2′-deoxyguanosine, Ototoxicidade induzida pela cisplatina, Eugenol, EOAPD, Estresse oxidativo, 8-Hidroxi-2′-deoxiguanosina

## Abstract

**Introduction:**

Ototoxicity refers to cellular damage or function impairment developing in the inner ear in association with any therapeutic agent or chemical substance, and still represents the principal side-effect restricting the use of cisplatin.

**Objective:**

The aim of this study was to perform a biochemical, functional and histopathological investigation of the potential protective effect of eugenol against cisplatin-induced ototoxicity.

**Methods:**

The study was performed with 24 female Sprague Dawley rats. Distortion product otoacoustic emissions tests were performed on all animals, which were randomized into four equal groups. A single intraperitoneal dose of 15 mg/kg cisplatin was administered to cisplatin group, while the eugenol group received 100 mg/kg eugenol intraperitoneal for five consecutive days. 100 mg/kg eugenol was administered to cisplatin + eugenol group for 5 days. On the third day, these rats were received a single dose of 15 mg/kg cisplatin. The control group was given 8 mL/kg/day intraperitoneal saline solution for five days. The distortion product otoacoustic emissions test was repeated 24 h after the final drug administration. All animals were sacrificed, and the cochleas were subsequently used for biochemical and histopathological examinations.

**Results:**

Cisplatin caused oxidative stress in the cochlea, impaired the cochlear structure and significantly reduced signal noise ratio levels. Administration of eugenol together with cisplatin reversed these effects and provided functional, biochemical and histopathological protection.

**Conclusion:**

The study findings represent the first indication in the literature that eugenol may protect against ototoxicity by raising levels of antioxidant enzymes and lowering those of oxidant parameters.

## Resumo

**Introdução:**

A ototoxicidade refere-se ao dano celular ou comprometimento da função da orelha interna associado a qualquer agente terapêutico ou substância química e ainda representa o principal efeito colateral que restringe o uso da cisplatina.

**Objetivo:**

O objetivo deste estudo foi realizar uma investigação bioquímica, funcional e histopatológica do potencial efeito protetor do eugenol contra a ototoxicidade induzida pela cisplatina.

**Método:**

O estudo foi realizado com 24 ratos fêmeas *Sprague Dawley*. Testes de emissões otoacústicas por produto de distorção foram realizados em todos os animais, os quais foram randomizados em quatro grupos iguais. Uma única dose intraperitoneal de 15 mg/kg de cisplatina foi administrada ao grupo cisplatina, enquanto o grupo eugenol recebeu 100 mg/kg de eugenol intraperitoneal por cinco dias consecutivos. Foram administrados 100 mg/kg de eugenol ao grupo cisplatina + eugenol durante 5 dias. No terceiro dia, estes ratos receberam uma dose única de 15 mg/kg de cisplatina. O grupo controle recebeu 8 mL/kg/dia de solução salina intraperitoneal por cinco dias. O teste de emissões otoacústicas por produto de distorção foi repetido 24 horas após a administração final do medicamento. Todos os animais foram sacrificados e as cócleas foram posteriormente utilizadas para exames bioquímicos e histopatológicos.

**Resultados:**

A cisplatina causou estresse oxidativo na cóclea, prejudicou a estrutura coclear e reduziu significativamente os níveis da relação sinal/ruído. A administração de eugenol juntamente com a cisplatina reverteu esses efeitos e forneceu proteção funcional, bioquímica e histopatológica.

**Conclusão:**

Os achados do estudo representam a primeira indicação na literatura de que o eugenol pode proteger contra a ototoxicidade, eleva os níveis de enzimas antioxidantes e diminui os níveis dos parâmetros oxidantes.

## Introduction

Cancer is one of the leading causes of death worldwide and a source of severe social disruption. The prevalence of cancer is rising all the time, thus resulting in increased use of chemotherapeutic agents.^1^ One important chemotherapeutic agent is platinum-containing cisplatin (cis-diamminedichloroplatinum II). This molecule consists of a divalent Pt central atom together with four ligands of cis-positioned pairs of chlorine atoms or amine groups.^1^ Since its discovery it has been employed to treat various types of cancer. However, side-effects such as nephrotoxicity, neurotoxicity and ototoxicity severely limit its use. Nephrotoxicity can be prevented by means of saline hydration and mannitol therapy, but neurotoxicity and ototoxicity still represent restricting factors.

Ototoxicity refers to inner ear damage characterized by transient or permanent hearing loss. Cisplatin-induced ototoxicity generally manifests in the form of irreversible, progressive, and bilateral sensorineural hearing loss, particularly at high frequencies. This loss may rarely be unilateral or asymmetrical. The degree of hearing loss is dose-dependent and is also related to the frequency and method of application.^2^ Since this clinical manifestation results in severe disturbances of quality of life and social communication, various agents have been used to prevent cisplatin-induced ototoxicity. The most desirable characteristic of such agents is that they should be capable of reducing the ototoxic effect of cisplatin while preserving its antitumor effectiveness. Vitamin E, N-acetylcysteine, dexamethasone, resveratrol and whortleberry have all been employed for this purpose.^3^, ^4^, ^5^, ^6^, ^7^ However, none has emerged as indisputably protective against cisplatin-induced ototoxicity. There is also no agent currently recommended for routine use.

Eugenol is a yellowish oily fluid obtained from the clove plant (Eugenia caryophyllata), which is endemic in various parts of the world. The oil has been shown to exhibit antimicrobial, analgesic, anti-inflammatory, antioxidant, anti-mutagenic, and anti-carcinogenic effects.^8^ The antioxidant activity of eugenol compounds derives from their methoxy-phenolic structure.^9^ Studies have shown that the antioxidant activity of eugenol protects against oxidative tissue damage in a range of experimental models. Eugenol is non-toxic, non-mutagenic, and non-carcinogenic, and is considered safe by the U.S. Food and Drug Administration.^10^

Our scan of the literature revealed no previous studies of the effect of eugenol on ototoxicity induced by cisplatin. The aim of this study was therefore to perform a biochemical, functional and histopathological investigation of whether eugenol exhibits a protective effect against cisplatin-induced ototoxicity.

## Methods

The study was approved by the local ethical committee of Ataturk University (n° E.1700283394-11:142). The principles of the Care and Use of Laboratory Animals Guideline were applied throughout the study. Twenty-four female Sprague Dawley rats weighing 250–300 g were housed in special cages with ad libitum access to chow and water throughout the experiment. The cages were kept in a room equipped with a fully automatic lighting system set to a 12 h light/12 h dark cycle. Temperature was fixed at 23 ±2 °C and humidity at 45%–50%.

Eugenol was supplied by the Sigma–Aldrich Chemical Co., and cisplatin from Kocak Farma Co. (Kocak, Istanbul, Turkey). At the beginning of the study, an anesthesia mixture was prepared with ketamine hydrochloride (Ketasol 10%, Richter Pharma Ag, Wels, Austria) and xylazine (Alfazyne 2%, Alfasan International BV, Voerden, Netherlands) at doses of 40 mg/kg and 5 mg/kg, respectively and administered intraperitonealy to all rats for otoscopic examination. The Distortion Product Otoacoustic Emissions (DPOAE) test was then carried out using appropriate probes. The 24 rats were then randomized into four groups of six animals each. An experimental ototoxicity model was established with a single Intraperitoneal (ip) dose of 15 mg/kg cisplatin in the cisplatin group (Group Cis). The eugenol group (Group E) received 100 mg/kg eugenol ip on five consecutive days. The cisplatin + eugenol group (Group CE) received 100 mg/kg eugenol ip for five consecutive days. On the third day of the study, these rats also received a single ip infection of 15 mg/kg cisplatin. The control group (Group C) was given 8 mL/kg/day ip saline solution for five days. The second DPOAE test was applied 24 h after the final drug administration, again under ketamine + xylazine anesthesia. All animals were sacrificed after the test. The cochleas of all rats were removed after the dissection of the temporal bones. The left cochleas were set aside for histopathological study and the right cochleas for biochemical analysis.

### DPOAE measurement

The test was performed using an Otometrics Madsen Capella device after the otoscopic examination of the rats under general anesthesia. Prior to each test, the probe was calibrated with an automated system and placed against the external ear canal. DPOAE measurement of the bilateral ears was then carried out in a silent environment using appropriate probes. Measurements consisted of DPgrams. Primary stimuli intensities of L1 = 65 dB and L2 = 55 dB were specified. Two frequencies, f1/f2 = 1.22, were employed in order to elicit the most powerful response. DPgram measurements were carried out at 8 frequencies between 2002 and 10 000 Hz. Signal Noise Ratio (SNR) values equal to or greater than 3 dB were assumed to represent positivity.

### Biochemical analysis

Following removal of the right cochleas, these were washed with physiological saline solution and stored at −80 °C until analysis. On the day of study, each cochlear tissue was ground in liquid nitrogen using a commercial Tissue Lyser II grinding jar kit (Qiagen, Hilden, Germany). All tissue specimens were homogenized in KCl buffer 900 μL 0.1 M KCl per 0.1 g tissue. Supernatant was obtained by centrifuging the homogenate at 4 °C for 30 min at 13 000 rpm. Malondialdehyde (MDA) levels in the homogenate were determined spectrophotometrically with the presence of thiobarbituric acid, using the method described by Ohkawa et al.^11^ Values were expressed as nmoL/mL. Superoxide Dismutase (SOD) activity was determined using the method described by Sun et al.^12^ and was expressed as U/mL. Glutathione peroxidase (GPx) activity was also determined spectrophotometrically following the technique described elsewhere by Paglia and Valentine,^13^ and concentrations were expressed as IU/L.

### Histopathological analysis

For histopathological evaluation, the left cochlear tissues were fixed in 10% formalin solution for 48 h. After softening for 96–120 h in Osteosoft (Merc, HC313331, Germany) solution for decalcification, they were washed in running water for 24 h. Before being embedded in paraffin blocks, tissues were passed through a series of alcohol and liquid paraffin. Sections 4 μm in thickness were collected from each block and transferred onto glass slides. Preparates were next stained with Masson's trichrome and photographs were taken. The presence of lesions was evaluated under light microscopy on a semi-quantitative scale of none (−), slight (+), moderate (++) and severe (+++). In semi-quantitative histopathological examination; hyperemia in the stria vascularis was classified as a vessel diameter ≤0.4–1 μm (−), 1–2 μm (+), 3–5 μm (++), and ≥5 μm (+++). Degeneration in the spiral ganglia was defined as a degenerated cell number of 0 (−), 3–5 (+), 6–10 (++), and ≥10 (+++). Structural impairment and decreased cell numbers in outer hair cells were defined as an impaired cell number of 0 (−), 3–5 (+), 6–10 (++), and ≥10 (+++).

### Immunohistochemical examination

For immunoperoxidase analysis, adhesive slides (poly-l-lysine) were used. After the transfer of sections to these slides, they were passed through xylol and alcohol series, prior to deparaffinization and drying. They were then washed in distilled water for 5 min. Sections were exposed to heat in a microwave, four times for 5 min each, in antigen retrieval (citrate buffer, pH 6.1) in order to prevent core antigen masking. They were then removed from the microwave and left to cool for 30 min at room temperature. Following these procedures they were washed with distilled water and dried. A glass pen was then used to delineate the areas surrounding the section of interest. They were washed for 5 min in Phosphate Buffer Solution (PBS) which have a pH value of 7.2 and kept in 3% H_2_O_2_ for 10 min for inactivation of endogenous peroxidase. Following washing in PBS for 5–10 min, specimens were incubated for 5 min with protein block compatible with all primary and secondary antibodies for the prevention of non-specific background staining. Following incubation, the excess block solution was removed from the slide surfaces. Primary antibodies PBS in the control group and 8-hydroxy-2′-deoxyguanosine (8-OHdG) were placed onto the sides without the application of a washing procedure. Depending on the primary antibody concerned, they were then kept either for 1 h at room temperature or overnight at +4 °C. They were washed twice for 5 min with PBS and incubated for 10–30 min at room temperature with biotinylated secondary antibodies. Sections were again washed with PBS and kept in streptavidin–peroxidase for 10–30 min. They were subsequently washed again with PBS in the same manner. In the next stage, 3-3′ diaminobenzidine (DAB) chromogen was dropped onto the sections and left in situ for 5–10 min for chromogen absorption. Background staining was provided by keeping the specimens 1–2 min in Mayer's hematoxylin. The specimens were then washed under tap water and subsequently passed through alcohol and xylol series, before being covered and subjected to analysis by means of a light microscope (Leica DM 1000). Immune positivity was graded as none (–), slight (+), moderate (++) or severe (+++) which was defined as a positive cell number of 0 (−), 3–5 (+), 6–10 (++), and ≥10 (+++).

### Statistical analysis

Statistical analysis was performed by using SPSS 17.0 software. Analysis of DPOAE measurements and biochemical values was performed using the Shapiro–Wilk test and histograms. One-way ANOVA was used for comparisons when normal distribution was established, and the Kruskal–Wallis test in case of non-normal distribution. Analysis of variations among data elicited by means of semi-quantitative histopathological examination was also performed using the Kruskal–Wallis test. The Tukey test was employed for post hoc analysis when ANOVA was applied and the Mann Whitney *U* test for two-way comparisons when significant differences were determined with the Kruskal–Wallis test. For all tests, *p* < 0.05 was regarded as statistically significant.

## Results

**Figure 1:**
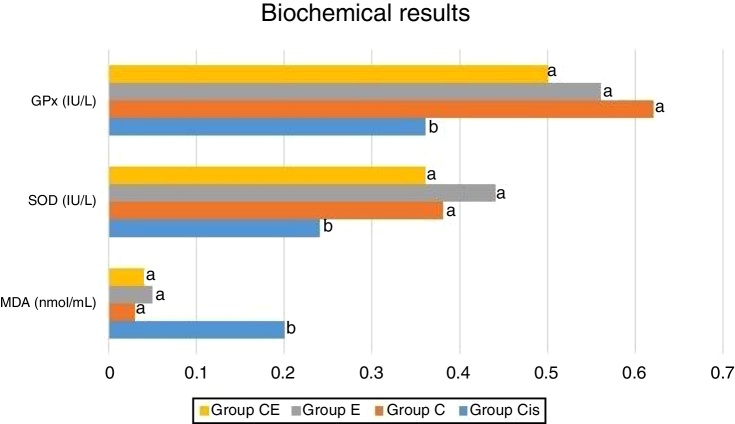
Biochemical results. The letters near each bar indicate the statistical comparison. Different letters indicate a significant difference at the *p* < 0.05 level, while the same letters indicate no significant difference.

Biochemical analysis of the rat cochleas revealed a significant increase in MDA levels in the cisplatin group compared to the control group, while significant decreases were observed in SOD and GPx activities. This shows that cisplatin causes oxidative stress in the cochlea and that oxidative stress may be one of the mechanisms involved in ototoxicity. The levels of MDA as well as the activities of SOD and GPx were similar in the eugenol and control groups. MDA levels were significantly lower in the cisplatin plus eugenol compared to the cisplatin only group. We also determined no significant difference in MDA levels of cisplatin plus eugenol group compared to the control group. Similarly, SOD and GPx activities in cisplatin plus eugenol group were found to be significantly higher than cisplatin only group, but no significant difference was determined between cisplatin plus eugenol and control groups. These findings show that cisplatin causes oxidative stress in the rat cochlea, but that administration of eugenol prevents oxidative stress. The biochemical analysis results are shown in Fig. 1.

**Figure 2:**
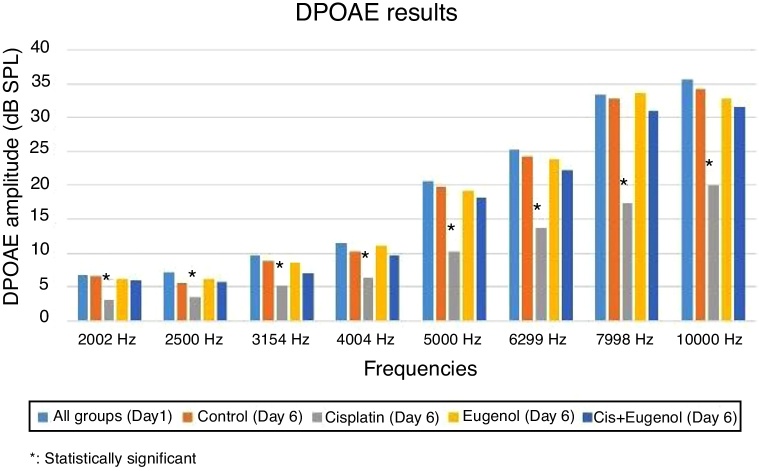
DPOAE results.

No significant variation was determined among SNR values in all DPgrams on the first day of the study. There was also no significant difference between SNR values of right and left ears in all groups on days 1 and 6. Comparison of the test results on the sixth day with those of the first day revealed a significant decrease in SNR values in the cisplatin group. Cisplatin group SNR values on the sixth day were significantly lower than those of the control group. This shows that cisplatin leads to functional damage in the rat cochlea. There was no significant difference between the SNR values of the rats treated with eugenol and either the control group or the first day values. No significant difference was also observed between the SNR values of the rats administered cisplatin together with eugenol and either the control group or the first day values. In addition, the SNR values of the cisplatin plus eugenol group were significantly higher than those of the rats receiving cisplatin only. These findings indicate that with its ototoxic effect, cisplatin reduced SNR values at all frequencies but that the administration of eugenol in combination with cisplatin prevented this decrease in SNR values. The DPOAE results are summarized in Fig. 2.

**Figure 3:**
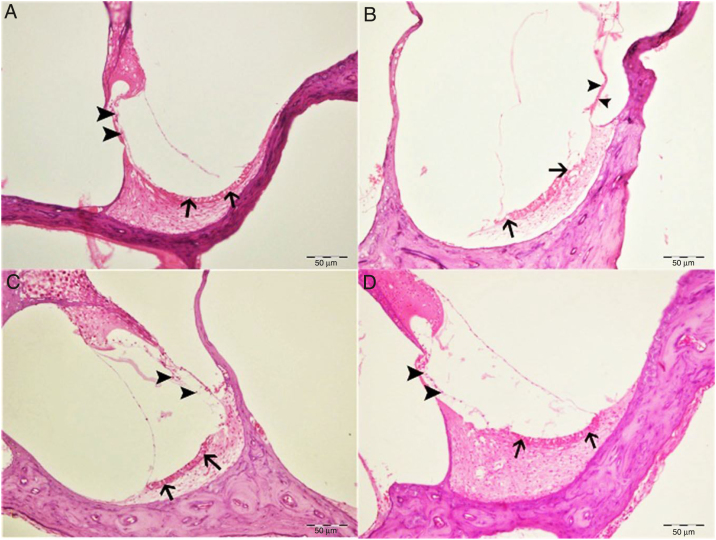
Histopathologic appearance of the cochlea, H&E, bar: 50 μm. (A) Control group. Normal histopathological structure of the cochlea (arrow, stria vaskularis; arrow head, outer hair cells). (B) Cisplatin group. Hyperemia, degeneration and erosion in the stria vascularis (arrows), severe decrease in the number of outer hair cells (arrow heads). (C) Eugenol group. Normal histopathological structure of the cochlea (arrow, stria vaskularis; arrow head, outer hair cells). (D) Cisplatin + eugenol group: mild hyperemia in the stria vaskularis (arrows), normal histopathological structure of outer hair cells with mild decrease in the number of these cells (arrow head).

Histopathological examination revealed normal architecture in the organ of Corti, stria vascularis and spiral ganglia in both the control and eugenol groups (Fig. 3A–C). However, the cisplatin group exhibited significant erosion in the stria vascularis together with degeneration and an edematous appearance in the connective tissue layer of endothelial cells. Spiral ganglion nerve cells were also severely degenerated. Impaired morphology was observed in the outer hair cells of the organ of Corti, and these cells had also significantly decreased in numbers due to degeneration and necrosis (*p* < 0.05) (Fig. 3B). Significantly fewer of these degenerative and necrotic findings were observed the Cis + eugenol group (*p* < 0.05), and eugenol appeared to provide significant protection against them (Fig. 3D). Histopathological findings in cochlear tissues from the different groups are summarized in Table 1.

**Table 1:** Histopathologic findings of the cochlea.

	Control group	Eugenol group	Cisplatin group	Cis + eugenol group
Hyperemia, degeneration and necrosis in stria vaskularis	–	–	+++	++
Degeneration in spiral ganglia	–	–	+++	+
Decrease in the number of outer hair cells with structural deformation	–	–	+++	+
Immunopositivity of 8-OHdG	–	–	+++	+

**Figure 4:**
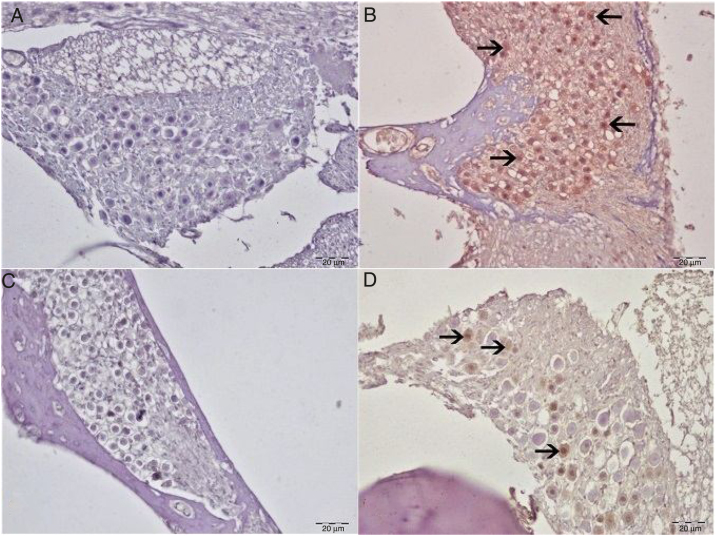
Immunohistochemical examination of the cochlea, IP, bar: 50 μm. (A) Control Group. Negative 8-OHdG expression in spiral ganglia. (B) Cisplatin group. Severe 8-OHdG immunopositivity in spiral ganglia (arrow). (C) Eugenol group: negative 8-OHdG expression in spiral ganglia. (D) Cisplatin + eugenol group: mild 8-OHdG immunopositivity in spiral ganglia (arrow).

8-Hydroxy-2′-deoxyguanosine (8-OHdG) staining was performed in order to identify oxidative DNA damage from the immunohistochemical perspective. No 8-OHdG immunopositivity was observed in either the control or eugenol groups (Fig. 4A–C). Severe immunopositivity was determined in spiral ganglion cells and outer hair cells in the cisplatin group (Fig. 4B). In the Cis + eugenol group, slight 8-OHdG expression was observed in spiral ganglion cells and outer hair cells (Fig. 4D). There was a significant decrease in 8-OHdG expressions between Cis + Eugenol and cisplatin groups (*p* < 0.05).

## Discussion

Ototoxicity describes cellular damage or function impairment occurring in the inner ear in association with any therapeutic agent or chemical substance. One of the therapeutic agents implicated in ototoxicity is cisplatin, widely employed as a chemotherapeutic agent. Recent studies have reported that cisplatin exhibits its cytotoxic effect through both nuclear and cytoplasmic signaling pathways.^14^ Molecular mechanisms capable of fully accounting for the cytotoxic effect of cisplatin have yet to be identified. However, studies have implicated increased Reactive Oxygen Species (ROS) production, a decrease in the antioxidant system, DNA damage, oxidative changes in proteins and increased lipid peroxidation in the development of ototoxic effects.^15^ We employed 8OHdG staining in order to reveal oxidative DNA damage from the immunohistochemical pespective. Severe immunopositivity was identified in spiral ganglion cells and outer hair cells in the cisplatin group, indicating the presence of oxidative stress.

The cochlea has a complex structure containing several cisplatin-sensitive cell types. Cisplatin-induced ototoxic effects manifest with damage in outer hair cells, the spiral ligament, support cells, and stria vascularis and spiral ganglion cells. The structure most sensitive to cisplatin in the inner ear is the hair cell. The outer hair cells in the basal turn of the cochlea are the most affected cells. This explains why the hearing loss that occurs in cisplatin-induced ototoxicity particularly involves high frequencies. The damage may also come to involve lower frequencies as treatment continues.^1^ In our study, histopathological examination of the cochlea in the cisplatin group revealed erosion in the stria vascularis, degeneration and edema in the connective tissue layer in endothelial cells, severe degeneration in spiral ganglion nerve cells, impaired morphology in the outer hair cells of the organ of Corti, together with a decrease in the numbers of these cells due to degeneration and necrosis.

The cochlea possesses various defense mechanisms against ototoxic effects, the most important of which are antioxidant enzyme systems such as SOD, catalase, and GPx. Protective systems such as adenosine receptors; heat shock proteins and hemooxygenase-1 are also present. However, the protective effect of these mechanisms is limited, and cell death occurs when the ototoxic effect overcomes the endogenous defense system.^16^ Biochemical examination in our study showed elevated MDA levels and decreased GPx and SOD activities in cochlear tissues of rat received cisplatin which also showed the impairment of oxidative balance.

Cisplatin-induced ototoxicity exhibits clinical effects in the early phase. In clinical terms, ototoxicity manifests with symmetrical, bilateral, progressive, irreversible and dose-dependent sensorineural hearing loss findings. DPOAE, an objective, rapid and non-invasive technique, is a test widely used to show this hearing loss in experimental studies. Since DPOAE reflects the activity of outer hair cells, it is effective even in showing damage occurring in the early stages. We used the DPOAE test in this study to show functional damage arising in the cochlea. The test was applied twice to all rats, on days 1 and 6. At all frequencies, the SNR values were significantly lower in rats received cisplatin than both day 1 SNR values and those of the control group. This shows that with its ototoxic effect, cisplatin caused hearing loss at all frequencies studied.

Since ototoxicity is the most important side-effect limiting the use of cisplatin in clinical practice, studies have investigated the potential otoprotective effects of various drugs. An ideal otoprotective agent must provide reliable protection while not compromising the antitumoral effect of cisplatin. It must also have minimal side-effects and be easy to apply. The agents that have received the greatest attention in that context are antioxidants that eliminate the effects of ROS. The present study investigated the otoprotective effect of eugenol, with known powerful antioxidant properties.

Eugenol is a yellowish oily fluid obtained from the clove plant which grows in various parts of the world. The oil has been shown to exhibit antimicrobial, analgesic, anti-inflammatory, antioxidant, antimutagenic, and anticarcinogenic effects.^8^ It was shown that, usage of eugenol together with cisplatin do not have a negative drug interaction. Thus, in a study performed by Islam et al., it was suggested that, in the combination of eugenol and cisplatin, eugenol potentiates the anticarsinogenic properties of cisplatin by inhibiting NF-κB pathway.^17^ Rao et al. investigated the effect of eugenol against cisplatin-induced nephrotoxicity and determined that it protects against cytotoxicity through free radical scavenging activities.^18^ Binu et al. investigated the therapeutic effect of eugenol against arsenic-induced cardiotoxicity. They reported that arsenic increased levels of MDA, a marker of lipid peroxidation, through its cytotoxic effect, and reduced levels of GSH, the most important non-enzymatic endogenous antioxidant, in heart tissue. Additionally, they observed that eugenol administered orally at 5 mg/kg increased tissue antioxidant levels, regulated membrane peroxidation and normalized heart rate. At the same time, they reported that MDA levels decreased as a result of eugenol therapy, while GSH and GPx levels increased. With these properties, they suggested that eugenol is a cytoprotective agent that prevents lipid peroxidation and that protects enzymatic and non-enzymatic antioxidant systems.^19^ Said et al. investigated the protective efficacy of eugenol in aluminum-induced brain damage and described eugenol as a potential neuroprotective agent due to its antioxidant and anti-apoptotic characteristics.^20^ Herrera et al. investigated the effectiveness of eugenol against gingival inflammation and reported anti-inflammatory effects when administered at low doses.^21^ In the present study, we observed an increase in MDA levels in cochlear tissue in cisplatin-induced ototoxicity and a decrease in GPx and SOD activities. Eugenol also reversed this effect, preventing oxidative stress and exhibiting otoprotective activity. We also determined no significant variation following the DPOAE test between the SNR values of rats receiving eugenol together with cisplatin and control group SNR values and day 1 SNR values. These findings show that eugenol prevents cisplatin-related cochlear damage.

## Conclusion

In conclusion, our findings demonstrate, for the first time in the current literature, that eugenol may protect against ototoxicity by raising levels of antioxidant enzymes and lowering those of oxidant parameters. We suggest that eugenol may be a suitable drug option against cisplatin ototoxicity.

## Ethical approval

This study was performed in accordance with the PHS Policy on Humane Care and Use of Laboratory Animals, the NIH Guide for the Care and Use of Laboratory Animals, and the Animal Welfare Act (7 U.S.C. et seq.); the animal use protocol was approved by the Institutional Animal Care and Use Committee (IACUC) of Ataturk University.

## Conflicts of interest

The authors declare no conflicts of interest.
